# Socioeconomic Gradients and Distribution of Diabetes, Hypertension, and Obesity in India

**DOI:** 10.1001/jamanetworkopen.2019.0411

**Published:** 2019-04-05

**Authors:** Daniel J. Corsi, S. V. Subramanian

**Affiliations:** 1 OMNI Research Group, Ottawa Hospital Research Institute, School of Epidemiology and Public Health, University of Ottawa, Ottawa, Ontario, Canada; 2 Department of Social and Behavioral Sciences, Harvard T.H. Chan School of Public Health, Boston, Massachusetts

## Abstract

**Importance:**

Cardiovascular disease and risk factors represent a major and increasing burden of death and disability in India, although socioeconomic aspects have been debated in recent years.

**Objective:**

To conduct a comprehensive equity analysis of the socioeconomic gradients and distribution of diabetes, hypertension, and obesity in India using the latest national data set.

**Design, Setting, and Participants:**

Cross-sectional study of data originating from the fourth Indian National Family Health Survey collected from January 20, 2015, to December 4, 2016. The study population was based on a nationally representative cross-sectional sample of women aged 15 to 49 years and men aged 15 to 54 years in India, with a response rate of 97% and 92% among eligible women and men, respectively. Biomarker sampling of survey respondents captured height, weight, blood pressure, and random blood glucose levels. Markers of socioeconomic status (SES) were household wealth, education, and social caste. Descriptive analyses and logistic regression models that account for multistage survey design and sampling weights were estimated.

**Main Outcomes and Measures:**

Diabetes, hypertension, and obesity assessed by predetermined thresholds based on biomarker sampling or current medication were the primary outcomes.

**Results:**

The survey covered 757 958 individuals (weighted prevalence of 51.2% female). The overall prevalence of diabetes, hypertension, and obesity in the sample was 2.9%, 14.4% and 9.7%, respectively. Positive socioeconomic gradients were observed by household wealth, education, and social caste, and in a majority of states. The magnitude of the SES gradient was strongest for obesity (adjusted odds ratio for highest SES quintile vs lowest, 8.76; 95% CI, 7.70-9.95), followed by diabetes (adjusted odds ratio, 2.31; 95% CI, 1.88-2.85) and hypertension (adjusted odds ratio, 1.58; 95% CI, 1.45-1.72) (*P* < .001 for all associations). Analyses of the socioeconomic distribution indicated that between 70% and 90% of the population burden of diabetes, hypertension, and obesity was among the higher SES groups, and this figure was similar across states.

**Conclusions and Relevance:**

Cardiovascular risk factors have an uneven distribution in India. Prevention and treatment strategies should reflect the distribution of the risk factor burden.

## Key Points

**Question:**

What is the socioeconomic distribution of diabetes, hypertension, and obesity in India, and what are the socioeconomic gradients?

**Findings:**

In this cross-sectional study of data from the Indian National Family Health Survey of 757 958 individuals, the population burden of cardiovascular disease risk factors was concentrated among groups with higher socioeconomic status. Positive and significant socioeconomic gradients were observed for household wealth and obesity, diabetes, and hypertension.

**Meaning:**

In resource-limited settings, prevention and treatment of cardiovascular risk factors should be proportional to the burden within the population.

## Teaser

This cross-sectional study uses data from the Indian National Family Health Survey to assess the socioeconomic gradients and distribution of diabetes, hypertension, and obesity in the Indian population.

## Introduction

Cardiovascular disease (CVD) and associated risk factors, including type 2 diabetes, hypertension, and obesity, represent a major and increasing burden of death and disability in India.^1^ In 2016, the India State-Level Disease Burden Initiative, part of the Global Burden of Disease Study, reported that ischemic heart disease (the most common form of CVD) was the leading cause of disability-adjusted life-years (a measure of overall disease burden) after previously being ranked sixth in 1990.^2^ Diabetes was ranked the 13th leading cause of disability-adjusted life-years in 2016, increasing more than 170% since 1990. This trend indicates changes in the burden of premature mortality and morbidity away from communicable, maternal, and neonatal conditions and toward CVD and other noncommunicable diseases at an aggregate scale in India.^2^ Although CVD may be the overall leading cause of mortality, and risk factors such as diabetes are an important component of this overall burden, summary statistics such as the Global Burden of Disease mask large variation in the distribution of CVD and risk factors along socioeconomic status (SES) dimensions given the enormous variation that has been described in India.^3,4^

Assessment of social inequalities in the burden of CVD and risk factors has been central to the equity debate around focus of disease priorities in India.^3^ A key issue is determining whether communicable or noncommunicable diseases represent the majority of disease burden among the poorest population groups. Two approaches have been used for assessing socioeconomic inequality in CVD and risk factors.^5,6,7,8,9,10^ The first, based on socioeconomic gradients of disease, uses a prevalence-based approach in which differences in prevalence of CVD and risk factor levels are compared across SES groups. A second approach based on the socioeconomic distribution of disease assesses the proportion of the total burden of CVD or risk factors that is composed of different SES groups. In this study, we used both approaches to present a comprehensive equity analysis of the SES distribution of the burden of CVD risk factors in India among women aged 15 to 49 years and men aged 15 to 54 years with a focus on diabetes, hypertension, and obesity.

## Methods

### Data Source

This study was approved by the Ottawa Health Science Network Research Ethics Board. Survey participants provided informed oral consent prior to each interview or biomarker test. We followed the Strengthening the Reporting of Observational Studies in Epidemiology (STROBE) reporting guideline and the American Association for Public Opinion Research (AAPOR) reporting guideline for reporting the methodology of this cross-sectional survey.^11^ Data originated in the fourth Indian National Family Health Survey (NFHS-4), a national survey conducted in India between 2015 and 2016 by the Ministry of Health and Family Welfare. The survey was intended to represent the population of women of reproductive age (15-49 years) and their partners (15-54 years) and collected information on sociodemographic characteristics, household water and sanitation, child and adult health, and other related variables. In addition, the NFHS-4 included biomarker sampling for height, weight, blood pressure, and blood glucose level.

### Survey Design

The NFHS-4 was a 2-stage stratified survey designed to be nationally representative of the household population of women aged 15 to 49 years and men aged 15 to 54 years and covered all states and union territories in India.^12^ In addition, the NFHS-4 included slum populations in 8 major cities. The survey used the 2011 Census of India as the sampling frame, and stratification was done by urban and rural areas with additional stratification in rural areas based on the proportion of scheduled castes and scheduled tribes. Primary sampling units (PSUs) were defined as census enumeration blocks in urban areas and villages in rural areas. The PSUs were selected with a probability proportional to size within each stratum. Selected PSUs were visited by field teams who compiled lists of all residential households to serve as the sampling frame for the second survey stage. A fixed number of 22 households was then randomly selected within PSUs to be visited by survey teams. All women aged 15 to 49 years who resided or spent the previous night in selected households were eligible for participation in the women’s survey. In a random subsample of about 15% of households, all men aged 15 to 54 years who resided or spent the night in these households were eligible for the men’s survey. Survey questionnaires were administered by interviewers and responses recorded using electronic data capture and CAPI software (Creative Research Systems) to provide feedback and ensure robustness of data quality. In addition to survey questions, biomarker sampling was conducted on all eligible women and men and included measurements of height, weight, blood pressure, and random blood glucose level. Data were collected from January 20, 2015, to December 4, 2016, by 14 field agencies. The survey response rate was nearly 98% at the household level and was 97% and 92% among eligible women and men, respectively.

### Outcomes

Outcomes were diabetes, hypertension, and obesity. Diabetes was measured via random blood glucose test. We used cutoffs to classify diabetes based on a random glucose level greater than 200 mg/dL (to convert to millimoles per liter, multiply by 0.0555) if the respondent was not fasting and greater than 126 mg/dL if the respondent was fasting prior to the test.^13^ Respondents who reported taking diabetes medication were considered to be diabetic. The mean of 3 systolic blood pressure and diastolic blood pressure measurements was obtained from survey participants. Cutoff points of systolic blood pressure 140 mm Hg or greater or diastolic blood pressure 90 mm Hg or greater were used to define measured hypertension. In addition, individuals who reported taking blood pressure medication were considered hypertensive. Body mass index (calculated as weight in kilograms divided by height in meters squared) was calculated for survey respondents with valid height and weight measurements. Obesity was classified as BMI of 27.5 or greater based on risk of type 2 diabetes and cardiovascular disease in Asian populations.^14^

### Markers of SES

Multiple markers of SES^15^ were considered, including social caste, household wealth, and education. Household wealth was defined by an index based on indicators of asset ownership and housing characteristics, which were reported in the survey and verified by interviewers.^16^ This index has been developed and validated in a number of countries to be a robust measure of wealth consistent with measures of income and expenditure.^17^ The measure was constructed using indicators of housing characteristics (eg, type of windows and flooring and water and sanitation facilities) and assets (eg, ownership of home, car, computer, and mobile phone), which were weighted and combined using a principal component analysis procedure.^18^ Wealth was categorized into 5 quintiles from poorest to richest households based on the national distribution.^19^ Education was categorized in 5 levels by number of years completed (no education, primary, secondary, higher secondary, and college). Social caste is based on self-reports and categorized as general caste, scheduled caste, scheduled tribe, other backward classes (OBC), and no caste. Scheduled castes and scheduled tribes are considered lower status and socially marginalized by the government of India.^20^ Dichotomous indicators for higher and lower wealth (quintiles 1-2 vs quintiles 3-5), education (no education vs primary and above), social caste (scheduled castes and scheduled tribes vs others), and a combined marker for low SES on all 3 dimensions were created.

### Covariates

Respondent characteristics included age, sex, current smoking, current alcohol use, and urban or rural place of residence. Smoking and alcohol use were self-reported by survey respondents. Urban or rural area was captured based on the survey and census definitions.

### Statistical Analysis

Prevalence estimates of diabetes, hypertension, and obesity were calculated accounting for the survey design and sampling weights. Descriptive analyses using frequencies and proportions were conducted to quantify the distribution of higher and lower SES groups among individuals with diabetes, hypertension, and obesity. Logistic regression was used to assess socioeconomic gradients in outcomes, and models accounted for survey design characteristics and sampling weights. Unadjusted and adjusted odds ratios (ORs) and 95% confidence intervals were calculated. We examined interactions between age and SES markers for evidence of effect modification using 2-sided Wald tests with a level of significance of 5%. Stratified analyses were conducted to compare the socioeconomic distribution and gradients in outcomes across states (excluding union territories). State-specific analyses were summarized using medians, quartiles, and box plots. All analyses were conducted in Stata statistical software version 15.1 (StataCorp LLC).

## Results

The NFHS-4 survey (2015-2016) covered 811 808 adults (699 686 women aged 15-49 years and 112 122 men aged 15-54 years). From this population, we defined a sample of 757 958 adults (weighted prevalence of 51.2% female) who had complete, valid data from the biomarker sampling and complete data on covariates (Table). Exclusions included 32 428 pregnant women and a further 21 422 respondents (2.7%) with missing data on outcomes or covariates. Excluded respondents were more likely to be of higher education and household wealth, although no differences in age were noted. The overall prevalence of diabetes was 2.9% (95% CI, 2.7%-3.0%), the prevalence of hypertension was 14.4% (95% CI, 14.1%-14.6%), and the prevalence of obesity was 9.7% (95% CI, 9.4%-10.0%). The prevalence of all risk factors increased with age. Diabetes and hypertension prevalence were higher among men, while obesity was more common in women (Table). An examination of the age structure by SES indicated that compared with the richest households, the poorest groups were younger (mean age, 30.8 years vs 32.0 years; difference, 1.35 years; 95% CI, 1.15-1.54 years; *P* < .001) and had a greater proportion of individuals younger than 20 years of age (19.7% vs 14.3%; difference, 5.39%; 95% CI, 4.67%-6.11%; *P* < .001) (eTable 1 in the Supplement).

**Table.** Characteristics of the Study Population and Prevalence of Diabetes, Hypertension, and Obesity, India, 2015-2016^a^

Characteristic	No. (%)^b^	Diabetes		Hypertension		Obesity	
		No. (%)^b^	Rate per 100^c^	No. (%)^b^	Rate per 100^c^	No. (%)^b^	Rate per 100^c^
All India	757 958 (100)	15 852 (100)	2.9	101 175 (100)	14.4	67 200 (100)	9.7
Age, y							
<20	135 866 (17.2)	435 (2.0)	0.3	4915 (4.2)	3.5	2060 (3.8)	2.1
20-24	122 206 (15.5)	607 (3.0)	0.6	7212 (6.5)	6.1	4473 (7.2)	4.5
25-29	118 078 (15.1)	1008 (5.5)	1.0	10 571 (10.3)	9.9	8318 (12.7)	8.2
30-34	105 239 (13.7)	1626 (10.5)	2.2	13 966 (13.3)	14.0	11 578 (16.8)	11.9
35-39	100 569 (12.8)	2549 (15.0)	3.4	18 179 (16.9)	19.0	13 491 (18.4)	13.9
40-44	86 091 (11.2)	3714 (22.1)	5.6	20 066 (19.0)	24.3	13 263 (18.8)	16.2
≥45	89 909 (14.6)	5913 (41.8)	8.2	26 266 (29.7)	29.4	14 017 (22.4)	14.9
Sex							
Male	107 842 (48.8)	3125 (55.5)	3.3	19 587 (58.9)	17.3	7691 (40.9)	8.1
Female	650 116 (51.2)	12 727 (44.5)	2.5	81 588 (41.1)	11.5	59 509 (59.1)	11.2
Current smoker							
Male	28 707 (97.9)	888 (98.8)	3.8	5650 (98.3)	18.7	1693 (98.0)	6.5
Female	5755 (2.1)	158 (1.2)	2.2	919 (1.7)	14.8	400 (2.0)	6.1
Current alcohol use							
Male	34 056 (95.8)	1139 (96.7)	4.0	7839 (96.3)	21.8	2585 (96.5)	8.8
Female	16 357 (4.2)	293 (3.3)	3.2	3501 (3.7)	19.0	999 (3.5)	7.3
Wealth							
Poorest	140 743 (15.7)	1462 (8.0)	1.5	15 727 (12.0)	11.0	2569 (2.7)	1.7
Poorer	161 480 (19.2)	2144 (11.8)	1.8	19 687 (16.0)	12.0	6366 (7.7)	3.9
Middle	160 916 (21.2)	2927 (18.4)	2.5	21 155 (20.8)	14.1	12 048 (16.6)	7.6
Richer	151 727 (21.8)	4253 (27.0)	3.5	22 345 (24.8)	16.4	19 486 (30.3)	13.4
Richest	143 092 (22.2)	5066 (34.9)	4.5	22 261 (26.4)	17.1	26 731 (42.8)	18.6
Education							
No schooling	199 891 (20.2)	4343 (19.2)	2.7	32 673 (22.9)	16.3	13 784 (14.7)	7.0
Primary	94 929 (12.4)	2314 (14.7)	3.4	14 766 (14.4)	16.7	8335 (11.8)	9.2
Secondary	197 495 (26.7)	3830 (26.0)	2.8	23 654 (24.5)	13.2	16 530 (24.3)	8.8
Higher secondary	175 919 (25.8)	3508 (25.1)	2.8	19 828 (23.2)	12.9	17 608 (29.2)	10.9
College	89 724 (15.0)	1857 (15.0)	2.9	10 254 (15.0)	14.4	10 943 (20.1)	13.0
Social caste							
General caste	152 769 (22.4)	4024 (26.3)	3.4	22 464 (24.5)	15.7	20 178 (31.2)	13.5
Scheduled caste	132 687 (19.7)	2535 (17.6)	2.6	16 229 (18.9)	13.8	9500 (15.3)	7.5
Scheduled tribe	140 736 (9.4)	1990 (5.6)	1.7	20 394 (9.3)	14.3	6831 (4.2)	4.3
Other backward class	295 867 (44.1)	6374 (44.2)	2.9	36 122 (42.6)	13.9	26 633 (44.6)	9.8
No caste	35 899 (4.5)	929 (6.2)	4.0	5966 (4.8)	15.2	4058 (4.9)	10.5
Urban residence							
No	534 791 (64.5)	8920 (52.8)	2.3	68 766 (60.3)	13.4	33 792 (44.1)	6.6
Yes	223 167 (35.5)	6932 (47.2)	3.8	32 409 (39.7)	16.1	33 408 (55.9)	15.2

^a^ Data are from the fourth Indian National Family Health Survey. Counts are unweighted, with frequencies adjusted for survey weights.

^b^ Column percentages.

^c^ Row percentages.

### Socioeconomic Gradient

Among the SES markers, household wealth emerged with a clear positive gradient with diabetes, hypertension, and obesity. The prevalence of diabetes varied between 1.5% among the poorest households to 4.5% among the richest; hypertension, 11.0% to 17.1%; and obesity, 1.7% to 18.6% (Table). Associations between education and outcomes were less consistent but remained positive with a clear increasing trend for obesity, with prevalence increasing from 7.0% among those with no schooling to 13.0% among the college educated. Typically, scheduled castes and tribes had lower levels of diabetes, hypertension, and obesity compared with other social caste groups.

### Socioeconomic Distribution

**Figure 1.**
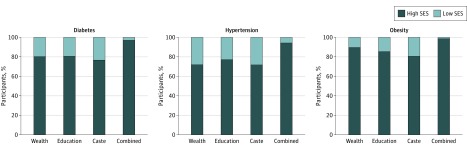
Distribution of Socioeconomic Status (SES) Among Individuals With Diabetes, Hypertension, or Obesity in India From 2015 to 2016 Data are from the fourth Indian National Family Health Survey. High and low SES groupings defined for wealth (quintiles 1-2 vs quintiles 3-5), education (no education vs primary and above), and social caste (scheduled castes and scheduled tribes vs others). Combined marker represents low SES on all 3 dimensions.

We examined the distribution of SES markers among individuals with diabetes, hypertension, and obesity. Using this metric, the distribution of wealth status among people with diabetes indicated that 80% had higher SES (wealth index quintiles 3, 4, and 5) compared with 20% with lower SES (wealth index quintiles 1 and 2). A total of 72% of individuals with hypertension and 90% of those with obesity were classified as having higher SES by wealth (Figure 1). The proportion of those with diabetes, hypertension, and obesity who were in the highest category of education varied from 77% for hypertension to 85% for obesity. The proportion of nonscheduled castes and tribes (higher SES) was 77%, 72%, and 81% among individuals with diabetes, hypertension, and obesity, respectively. The combined indicator of low SES by household wealth, education, and caste represented 6.0% of the survey population and 3.0%, 6.0%, and 1.4% of those with diabetes, hypertension, and obesity, respectively.

### Multivariable Analyses of Socioeconomic Gradients in CVD Risk Factors

**Figure 2.**
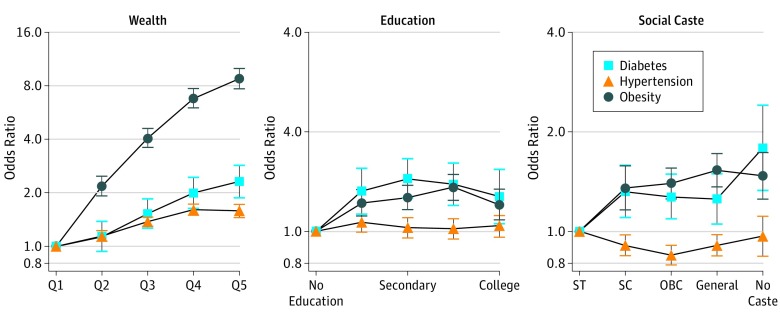
Adjusted Odds Ratios and 95% Confidence Intervals for the Social Gradients in Wealth, Education, and Social Caste for Diabetes, Hypertension, and Obesity in India From 2015 to 2016 Data are from the fourth Indian National Family Health Survey. OBC indicates other backward class; Q, quintile; SC, scheduled caste; and ST, scheduled tribe.

Logistic regression indicated that age was the strongest predictor of diabetes in unadjusted models, followed by household wealth (eTable 2 in the Supplement). The unadjusted OR for diabetes was 3.19 (95% CI, 2.67-3.83) among those from the richest compared with the poorest households. In adjusted models, the likelihood of having diabetes remained significantly increased for the highest quintile of household wealth compared with the poorest (OR, 2.31; 95% CI, 1.88-2.85) (Figure 2). Education was positively associated with diabetes in the adjusted model, although the strength of association did not increase with additional years. Primary, secondary, higher secondary, and college education were associated with increased odds for diabetes compared with no schooling (OR varying between 1.27 and 1.44), accounting for other factors. Compared with scheduled tribes, individuals with no caste had higher odds of diabetes in the adjusted model (OR, 1.79; 95% CI, 1.33-2.41). Age by wealth interactions indicated that the wealth gaps in prevalence of diabetes increased with age (*F*_24,28 415_ = 4.64; *P* < .001) (eFigure in the Supplement).

Wealth was positively associated with hypertension, and the direction and magnitude remained stable in the fully adjusted model (OR, 1.58; 95% CI, 1.45-1.72) (Figure 2). Secondary and higher education were inversely associated with hypertension in the unadjusted model, although the association was null after adjusting for other covariates. Compared with scheduled tribes, scheduled castes, OBC, and general castes had lower odds of hypertension in the adjusted model (eTable 3 in the Supplement). This association was equivalent to approximately 10% lower prevalence of hypertension among respondents of general caste (OR, 0.91; 95% CI, 0.84-0.98), and this difference was greater compared with OBC (OR, 0.85; 95% CI, 0.79-0.91). The interaction between age group and wealth index revealed statistically significant effect modification (*F*_24,28 415_ = 4.36; *P* < .001). The gradient in the prevalence of hypertension among the top 2 wealth quintiles appeared steeper at older ages.

The magnitude of the socioeconomic gradient was more pronounced for obesity. Wealth was robustly associated with obesity and the OR was 8.76 (95% CI, 7.70-9.95) for households in the richest quintile compared with the poorest after accounting for covariates (Figure 2). Education was significantly associated with obesity but the magnitude was much weaker, and, like diabetes, the association did not increase with further schooling in the mutually adjusted model (OR for secondary, higher secondary, and college varied between 1.20 and 1.36) (eTable 4 in the Supplement). Social caste had a positive association with obesity and the general caste had an OR of 1.53 (95% CI, 1.37-1.72) for obesity compared with scheduled tribes. The interaction between age group and wealth index was not statistically significant (*F*_24,28 415_ = 1.32; *P* = .14), and a plot of this association revealed that the wealth gradient in the prevalence of obesity was relatively stable with age.

### Variation in Socioeconomic Gradients and Distribution of CVD Risk Factors by State

**Figure 3.**
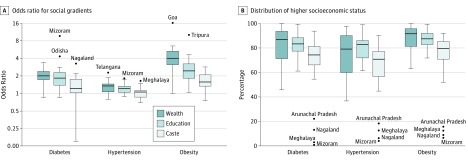
State-Specific Age-Adjusted Odds Ratios for Social Gradients and Distribution of Higher Socioeconomic Status The box plots show state-specific age-adjusted odds ratios for social gradients (A) and distribution of higher socioeconomic status (B) by wealth, education, and social caste for diabetes, hypertension, and obesity in India. The boxes indicate first and third quartiles with median lines at the center. Whiskers indicate first quartile − 1.5 × interquartile range and third quartile + 1.5 × interquartile range. Values outside the whiskers are shown as dots. Data are from the fourth Indian National Family Health Survey (2015-2016).

We examined the strength of the socioeconomic gradients in diabetes, hypertension, and obesity by household wealth, education, and social caste (Figure 3A). Across states, the median (interquartile range [IQR]) OR for diabetes among higher wealth compared with lower wealth was 2.0 (1.77-2.40) and was greater than 1.0 in 29 of 31 states (94%). The associations for education were similar (median [IQR] OR, 1.8 [1.38-2.29]; 97% positive) and weaker for caste (median [IQR] OR, 1.2 [1.02-1.76]) with 25 of 31 states (81%) having a positive gradient. Gradients for SES in hypertension were more variable between states, particularly for caste (median [IQR] OR, 1.06 [0.86-1.13]), with a positive gradient in 61% of states. The associations were smaller for wealth and education (median OR, 1.35 and 1.22, respectively) but remained positive in 87% and 90% of states, respectively. For obesity, SES gradients were positive and consistent across all states for wealth (median OR, 4.11) and education (median OR, 2.45) and for 97% of states for caste (median OR, 1.57). The median distribution of diabetes among higher SES groups based on wealth, education, and caste varied between 74% and 87% by state (Figure 3B). The distribution of higher SES groups was similar for hypertension (between 71% and 83%) and higher for obesity (between 79% and 92%). Four northeastern states (Arunachal Pradesh, Nagaland, Meghalaya, and Mizoram) had less than 25% of the burden of diabetes, hypertension, and obesity among the higher castes, and scheduled tribes composed the majority of population in these states.

## Discussion

This article provides a comprehensive picture of the socioeconomic gradients and distribution of diabetes, hypertension, and obesity in India using a recent national survey. We have several key findings. First, analyses of socioeconomic gradients by wealth, education, and social caste in the prevalence of diabetes, hypertension, and obesity were generally positive. The strongest and most consistent gradients were observed when using household wealth as the SES marker. The gradients were positive but of smaller magnitude for education and social caste. The magnitude of the gradient for each SES marker was strongest for obesity, followed by diabetes and hypertension. The socioeconomic gradient in hypertension was inverted for some categories of social caste. Analyses of the socioeconomic distribution indicated that between 70% and 90% of the population burden of diabetes, hypertension, and obesity was among higher SES groups, and this figure was consistent across states. A low SES population group made up of the lowest quintile of household wealth, those with no education, and those belonging to marginalized social castes or tribes accounted for between 1% and 6% of the burden of diabetes, hypertension, and obesity.

### Limitations

A limitation of the NFHS data is that, given the survey focus on maternal-child health and reproductive health in women, the survey is limited to age 49 years in women and 54 years in men. Thus, it appears that the prevalence of CVD risk factors that emerge after age 55 years may be underestimated in this data set, although CVD and risk factors appear to emerge up to 5 to 10 years earlier in South Asian individuals compared with other countries.^21^ All estimates have been age adjusted to account for age differences between male and female respondents. A recent analysis of the District Level Health Survey and the Annual Health Survey covering all adults aged 18 years and older^22^ reported a prevalence of diabetes and hypertension of 7.5% and 25.3%, slightly more than twice the prevalence reported here. For diabetes, our own estimate of the overall prevalence in India is approximately 7% using data from the Indian Council of Medical Research–India Diabetes study (data collected 2012-2015),^23^ NFHS (2015-2016), and the District Level Household and Facility Survey (2012-2013).^24^ All surveys were conducted within 4 years of each other and reported similar prevalence levels, with the NFHS having the lowest prevalence owing to the younger age of the sample. Prevalence estimates varied between 4% and 12% depending on cutoffs used and whether fasting blood samples were obtained. Self-reported prevalence data appears to be generally lower, perhaps owing to reduced awareness or diagnosis,^4^ and all data in the present study were focused only on the measured biomarkers for glucose and blood pressure. Further investigation is required to understand differences in prevalence estimates, and there may be differences between the surveys in sampling design, age adjustment and/or standardization, or method of testing.

The nature of large national surveys such as NFHS is that the diabetes assessment does not distinguish between type 1 and type 2 diabetes. Type 2 diabetes is the most common form of diabetes globally, accounting for more than 85% of cases.^25^ In addition, previous studies have focused on prediabetes, which may later develop into type 2 diabetes.^23^ Our analysis did not consider prediabetes, and it is possible that dietary and/or lifestyle interventions, in conjunction with medication such as metformin, may be able to limit such progression if identified early.

Concern has been raised over the anticipated rapid increase in diabetes and CVD risk factor prevalence in India. This increase and associated CVD-related morbidity and mortality are projected to coincide with reductions in deaths due to communicable diseases and maternal, perinatal, and nutritional causes, as has been described in the Global Burden of Disease Study.^2,26^ This contrasts with the situation in high-income countries, where CVD-related mortality has declined considerably since 1960.^27^ The prevalence of diabetes, hypertension, and obesity are also differentially distributed across the SES groups and geographic areas in India such that any increases will not be consistent across the population. It has been suggested that the prevalence of type 2 diabetes and other cardiovascular disease risk factors may increasingly become concentrated among low SES groups in India^28^ and other low- and middle-income countries,^29^ although to date, the empirical evidence in support of this hypothesis from national studies remains limited. Our findings clearly demonstrate that positive SES gradients remain across CVD risk factors and by different markers of SES. Interestingly, there was some evidence of an inverse gradient in hypertension among scheduled castes, OBC, and general castes when compared with scheduled tribes, although adjusted absolute difference in prevalence was less than 2%. Some recent studies have noted a high prevalence of hypertension in tribal populations in northeast India.^30,31^ A systematic review of primary hypertension among 53 tribal populations indicated a pooled prevalence of 16.1%, which was very close to our adjusted estimate of 15.7%.^32^ The best evidence on the secular increases in risk factor prevalence in India, however, has been limited to urban areas of southern India, which are more likely to be among the higher SES groups. Although the risk factor burden is greater among the higher SES groups, mortality is lower,^3^ suggesting that wealthier groups have better access to treatments and health care, possibly through private insurance or through greater affordability of out-of-pocket health expenditures.

To address this burden equitably, however, requires several considerations. Resource allocation should be optimized proportional to the burden of disease within states or districts. India’s Ministry of Health and Family Welfare has been establishing policies and strategies around the prevention and control of noncommunicable diseases in recent years, including, for example, The National Programme for Prevention and Control of Cancer, Diabetes, Cardiovascular Diseases and Stroke. In many districts and rural areas, however, the population continues to face a substantial burden of communicable diseases and maternal-child undernutrition.^33^ Continuing efforts are required to ensure progress on improving the social circumstances and conditions in these areas while also promoting improvements in health behaviors such as smoking and poor diet, which can improve the cardiovascular disease risk factor profile.

## Conclusions

As the burden of CVD and risk factors increase, health systems need to develop appropriate mechanisms to ensure access to detection, treatment, and control of CVD and risk factors across SES groups, for example, by increasing access to health workers and low-cost medication (eg, metformin or β-blockers) for diabetes and hypertension. India has experienced tremendous economic growth in recent decades, but improvements have been remarkably uneven and concentrated among a small minority.^34^ There is a risk that health care resources could follow a similar trajectory amid the target of universal health coverage.

## Notes

Supplement. **eTable 1.** Age Distribution of Survey Respondents by Household Wealth Quintile, Indian National Family Health Survey 2015-16 **eTable 2.** Unadjusted and Mutually Adjusted Odds Ratios and 95% Confidence Intervals From Logistic Regression Analyses With Diabetes as the Dependent Variable, India, NFHS-4 2015-2016 **eTable 3.** Unadjusted and Mutually Adjusted Odds Ratios and 95% Confidence Intervals From Logistic Regression Analyses With Hypertension as the Dependent Variable, India, NFHS-4 2015-2016 **eTable 4.** Unadjusted and Mutually Adjusted Odds Ratios and 95% Confidence Intervals From Logistic Regression Analyses With Obesity as the Dependent Variable, India, NFHS-4 2015-2016 **eFigure.** Adjusted Prevalence of Diabetes, Hypertension, and Obesity by Age for Quintiles of Household Wealth, India NFHS 2015-16
