# Supplementary material for: Socioeconomic Gradients and Distribution of Diabetes, Hypertension, and Obesity in India
Source: JAMA Netw Open. 2019 Apr 5;2(4):e190411. doi: 10.1001/jamanetworkopen.2019.0411 (PMC6450330; doi:10.1001/jamanetworkopen.2019.0411)
Supplement: Supplement. — eTable 1. Age Distribution of Survey Respondents by Household Wealth Quintile, Indian National Family Health Survey 2015-16 eTable 2. Unadjusted and Mutually Adjusted Odds Ratios and 95% Confidence Intervals From Logistic Regression Analyses With Diabetes as the Dependent Variable, India, NFHS-4 2015-2016 eTable 3. Unadjusted and Mutually Adjusted Odds Ratios and 95% Confidence Intervals From Logistic Regression Analyses With Hypertension as the Dependent Variable, India, NFHS-4 2015-2016 eTable 4. Unadjusted and Mutually Adjusted Odds Ratios and 95% Confidence Intervals From Logistic Regression Analyses With Obesity as the Dependent Variable, India, NFHS-4 2015-2016 eFigure. Adjusted Prevalence of Diabetes, Hypertension, and Obesity by Age for Quintiles of Household Wealth, India NFHS 2015-16 [file jamanetwopen-2-e190411-s001.pdf]

Supplementary Online Content

Corsi DJ, Subramanian SV. Socioeconomic gradients and distribution of diabetes, hypertension, and obesity in India. *JAMA Netw Open*. 2019;2(4):e190411. doi:10.1001/jamanetworkopen.2019.0411

eTable 1. Age Distribution of Survey Respondents by Household Wealth Quintile, Indian National Family Health Survey 2015-16
eTable 2. Unadjusted and Mutually Adjusted Odds Ratios and 95% Confidence Intervals From Logistic Regression Analyses With Diabetes as the Dependent Variable, India, NFHS-4 2015-2016
eTable 3. Unadjusted and Mutually Adjusted Odds Ratios and 95% Confidence Intervals From Logistic Regression Analyses With Hypertension as the Dependent Variable, India, NFHS-4 2015-2016
eTable 4. Unadjusted and Mutually Adjusted Odds Ratios and 95% Confidence Intervals From Logistic Regression Analyses With Obesity as the Dependent Variable, India, NFHS-4 2015-2016
eFigure. Adjusted Prevalence of Diabetes, Hypertension, and Obesity by Age for Quintiles of Household Wealth, India NFHS 2015-16

This supplementary material has been provided by the authors to give readers additional information about their work.

© 2019 Corsi DJ et al. *JAMA Network Open.*

eTable 1 Age distribution of survey respondents by household wealth quintile, Indian National Family Health Survey 2015-16

Age (years)	Poorest		Q2		Q3		Q4		Richest	
	No.	%	No.	%	No.	%	No.	%	No.	%
<20 y	28144	19.7	32431	19.4	29871	17.6	25247	16.2	20173	14.3
20-24 y	20402	14.0	26032	15.9	27048	16.1	25471	15.8	23253	15.1
25-29 y	21380	14.3	24132	14.5	25344	15.8	24112	15.3	23110	15.1
30-34 y	20146	13.8	21610	13.2	21842	13.4	21308	13.9	20333	14.0
35-39 y	19229	13.2	21057	12.7	20648	12.5	20269	12.8	19366	12.9
40-44 y	15647	11.2	17834	10.4	17715	10.7	17286	11.3	17609	12.5
45+ y	15795	13.9	18384	13.9	18448	13.9	18034	14.7	19248	16.1

© 2019 Corsi DJ et al. *JAMA Network Open.*

eTable 2 Unadjusted and mutually adjusted odds ratios and 95% confidence intervals from logistic regression analyses with diabetes as the dependent variable, India, NFHS-4 2015-2016

	**Unadjusted**				**Mutually Adjusted**		
	**OR**	**95% CI**			**OR**	**95% CI**	
Age (years)							
<20 y	1.00				1.00		
20-24 y	1.69	(1.23 -	2.32)		1.67	(1.22 -	2.31)
25-29 y	3.15	(2.31 -	4.28)		3.09	(2.27 -	4.21)
30-34 y	6.77	(5.13 -	8.93)		6.70	(5.07 -	8.86)
35-39 y	10.43	(8.00 -	13.60)		10.58	(8.10 -	13.84)
40-44 y	17.96	(13.75 -	23.46)		18.25	(13.94 -	23.89)
45+ y	26.95	(20.80 -	34.93)		27.70	(21.26 -	36.09)
Male	1.32	(1.22 -	1.42)		1.03	(0.93 -	1.15)
Current smokers	1.40	(1.25 -	1.56)		0.91	(0.80 -	1.03)
Current alcohol use	1.52	(1.38 -	1.68)		1.20	(1.07 -	1.36)
Wealth							
poorest	1.00				1.00		
poorer	1.21	(1.01 -	1.46)		1.13	(0.94 -	1.37)
middle	1.73	(1.45 -	2.07)		1.52	(1.26 -	1.84)
richer	2.49	(2.08 -	2.98)		2.00	(1.64 -	2.43)
richest	3.19	(2.67 -	3.83)		2.31	(1.88 -	2.85)
Education							
No schooling	1.00				1.00		
Primary	1.25	(1.08 -	1.46)		1.33	(1.13 -	1.55)
Secondary	1.02	(0.90 -	1.16)		1.44	(1.25 -	1.66)
Higher secondary	1.02	(0.90 -	1.16)		1.39	(1.20 -	1.61)
College	1.05	(0.89 -	1.23)		1.27	(1.05 -	1.54)
Social Caste							
General caste	2.00	(1.70 -	2.36)		1.25	(1.05 -	1.49)
Scheduled caste	1.51	(1.26 -	1.81)		1.32	(1.11 -	1.57)
Scheduled tribe	1.00						
Other backward class	1.69	(1.46 -	1.97)		1.27	(1.09 -	1.48)
No caste	2.38	(1.77 -	3.19)		1.79	(1.33 -	2.41)
Urban residence	1.65	(1.49 -	1.83)		1.12	(1.01 -	1.25)

© 2019 Corsi DJ et al. *JAMA Network Open.*

eTable 3 Unadjusted and mutually adjusted odds ratios and 95% confidence intervals from logistic regression analyses with hypertension as the dependent variable, India, NFHS-4 2015-2016

	**Unadjusted**				**Mutually Adjusted**		
	**OR**	**95% CI**			**OR**	**95% CI**	
Age (years)							
<20 y	1.00				1.00		
20-24 y	1.79	(1.62 -	1.97)		1.73	(1.57 -	1.91)
25-29 y	3.03	(2.75 -	3.33)		2.91	(2.64 -	3.20)
30-34 y	4.50	(4.12 -	4.93)		4.35	(3.97 -	4.77)
35-39 y	6.49	(5.95 -	7.07)		6.33	(5.79 -	6.92)
40-44 y	8.87	(8.13 -	9.67)		8.69	(7.95 -	9.51)
45+ y	11.50	(10.60 -	12.47)		10.81	(9.91 -	11.78)
Male	1.61	(1.55 -	1.66)		1.46	(1.39 -	1.52)
Current smokers	1.43	(1.36 -	1.50)		0.77	(0.73 -	0.82)
Current alcohol use	1.84	(1.75 -	1.93)		1.33	(1.26 -	1.41)
Wealth							
poorest	1.00				1.00		
poorer	1.10	(1.04 -	1.17)		1.14	(1.07 -	1.22)
middle	1.33	(1.25 -	1.41)		1.38	(1.29 -	1.47)
richer	1.58	(1.49 -	1.69)		1.60	(1.49 -	1.72)
richest	1.67	(1.56 -	1.78)		1.58	(1.45 -	1.72)
Education							
No schooling	1.00				1.00		
Primary	1.03	(0.97 -	1.10)		1.06	(1.00 -	1.14)
Secondary	0.78	(0.74 -	0.82)		1.03	(0.97 -	1.09)
Higher secondary	0.76	(0.72 -	0.81)		1.02	(0.96 -	1.08)
College	0.87	(0.81 -	0.93)		1.04	(0.96 -	1.12)
Social Caste							
General caste	1.12	(1.05 -	1.20)		0.91	(0.84 -	0.98)
Scheduled caste	0.96	(0.89 -	1.03)		0.90	(0.84 -	0.97)
Scheduled tribe	1.00				1.00		
Other backward class	0.97	(0.91 -	1.03)		0.85	(0.79 -	0.91)
No caste	1.08	(0.95 -	1.22)		0.97	(0.84 -	1.11)
Urban residence	1.24	(1.18 -	1.29)		1.03	(0.98 -	1.09)

© 2019 Corsi DJ et al. *JAMA Network Open.*

eTable 4 Unadjusted and mutually adjusted odds ratios and 95% confidence intervals from logistic regression analyses with Obesity as the dependent variable, India, NFHS-4 2015-2016

	**Unadjusted**				**Mutually Adjusted**		
	**OR**	**95% CI**			**OR**	**95% CI**	
Age (years)							
<20 y	1.00				1.00		
20-24 y	2.19	(1.78 -	2.69)		2.12	(1.72 -	2.60)
25-29 y	4.11	(3.35 -	5.04)		4.09	(3.34 -	5.00)
30-34 y	6.23	(5.24 -	7.41)		6.34	(5.33 -	7.55)
35-39 y	7.47	(6.26 -	8.92)		7.93	(6.64 -	9.49)
40-44 y	8.93	(7.43 -	10.74)		9.38	(7.79 -	11.28)
45+ y	8.08	(6.62 -	9.85)		9.21	(7.52 -	11.27)
Male	0.70	(0.67 -	0.74)		0.66	(0.62 -	0.70)
Current smokers	0.62	(0.57 -	0.68)		0.65	(0.59 -	0.72)
Current alcohol use	0.87	(0.81 -	0.93)		1.12	(1.03 -	1.21)
Wealth							
poorest	1.00				1.00		
poorer	2.35	(2.07 -	2.66)		2.18	(1.92 -	2.47)
middle	4.79	(4.26 -	5.39)		4.06	(3.59 -	4.58)
richer	9.07	(8.07 -	10.19)		6.80	(6.00 -	7.69)
richest	13.39	(11.93 -	15.03)		8.76	(7.70 -	9.96)
Education							
No schooling	1.00				1.00		
Primary	1.34	(1.22 -	1.47)		1.22	(1.11 -	1.34)
Secondary	1.28	(1.18 -	1.38)		1.26	(1.16 -	1.37)
Higher secondary	1.62	(1.50 -	1.76)		1.36	(1.24 -	1.49)
College	1.98	(1.79 -	2.18)		1.20	(1.08 -	1.34)
Social Caste							
General caste	3.48	(3.10 -	3.91)		1.53	(1.37 -	1.72)
Scheduled caste	1.82	(1.56 -	2.12)		1.35	(1.17 -	1.57)
Scheduled tribe	1.00				1.00		
Other backward class	2.42	(2.18 -	2.69)		1.40	(1.26 -	1.55)
No caste	2.61	(2.19 -	3.12)		1.47	(1.25 -	1.73)
Urban residence	2.54	(2.38 -	2.71)		1.33	(1.25 -	1.42)

© 2019 Corsi DJ et al. *JAMA Network Open.*

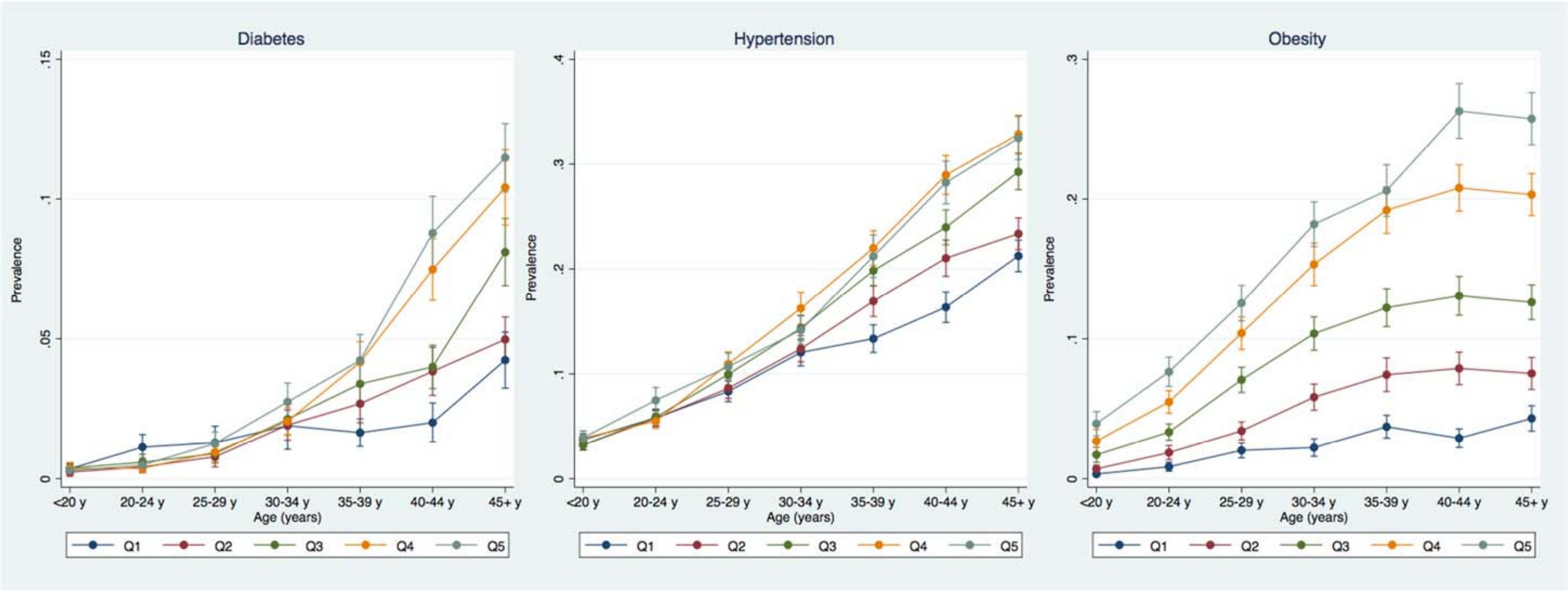

eFigure Adjusted prevalence of diabetes, hypertension, and obesity by age for quintiles of household wealth, India NFHS 2015-16.

Note: Adjusted prevalence calculated from mutually adjusted logistic regression model including an interaction effect for age group by wealth. Interaction p-values: diabetes (F(24, 28415)=4.64, p<0.001); hypertension (F(24, 28415)=4.36, p<0.001); obesity (F(24, 28415)=1.32, p=0.14)
